# DCIR in the “osteo-immune” system

**DOI:** 10.18632/oncotarget.6043

**Published:** 2015-10-09

**Authors:** Takumi Maruhashi, Tomonori Kaifu, Yoichiro Iwakura

**Affiliations:** Research Institute for Biomedical Sciences, Tokyo University of Science, Noda, Chiba, Japan

**Keywords:** C-type lectin, osteo-immune system, autoimmunity, bone metabolism, IFN-γ

Dendritic cell immunoreceptor (*Clec4a2,* DCIR) is one of C-type lectin receptors (CLRs), which is predominantly expressed in dendritic cells (DCs) [1]. DCIR contains a carbohydrate recognition domain in the extracellular part and an immunoreceptor tyrosine-based inhibitory motif (ITIM) in the cytoplasmic region which can negatively regulate immune signaling by recruiting phosphatases, SHP-1 and SHP-2. Previously, Fujikado et al. reported that *Dcir*^−/−^ mice spontaneously develop autoimmune sialadenitis and enthesitis, that eventually causes ankylotic changes of joints with age [2]. These mice are also highly susceptible to collagen-induced arthritis and experimental autoimmune encephalomyelitis, animal models for rheumatoid arthritis (RA) and multiple sclerosis, respectively. This is because antigen presentation is enhanced in *Dcir*^−/−^ mice, due to over-expansion of DC population. They showed that *Dcir*-deficient bone marrow cells differentiate into DCs more efficiently *in vitro* upon treatment with GM-CSF, because STAT5 activation is augmented in *Dcir*^−/−^ cells [2]. Thus, DCIR plays an important role in regulating homeostasis of the immune system by regulating DC development and expansion.

Recently, Maruhashi and Kaifu et al. investigated the mechanism how joint ankylosis is induced in *Dcir*^−/−^ mice [3]. They found that not only joint ankylosis but also mild bone volume increase of thigh bones occurs in *Dcir*^−/−^ mice. Interestingly, these abnormalities are completely abolished in *Dcir*^−/−^*Rag2*^−/−^mice and *Dcir*^−/−^*Ifng*^−/−^mice. IFN-γ-producing T cell population is increased in *Dcir*^−/−^ mice, and co-culture of purified *Dcir*^−/−^ DCs with wild-type T cells promotes differentiation of IFN-γ-producing T cells more efficiently than wild-type DCs, consistent with the report by Kaneko et al., in which they showed that DCs derived from SHP-1-deficient mice preferentially support IFN-γ-producing Th1 cell differentiation [4]. Furthermore, Maruhashi and Kaifu et al. showed that IFN-γ enhances proliferation and differentiation of chondrocytes and osteoblasts *in vitro*, suggesting that these osteogenic and chondrogenic activities of IFN-γ directly contribute to the bone abnormalities in *Dcir*^−/−^ mice [3]. These observations suggest that DCIR is critically involved in the regulation of bone homeostasis through regulation of IFN-γ-producing T cell differentiation via ITIM-SHP-1 activation. Thus, DCIR is important not only for immune homeostasis but also for bone metabolism, indicating that DCIR is one of regulators that coordinate bone formation and immune activation. As such, the immune and bone systems share a large number of regulatory components including cytokines, signaling molecules, transcriptional factors, and receptors [5]. However, the biological meaning of this coordination largely remains to be elucidated.

Ankylosing spondylitis (AS), a form of seronegative spondyloarthritis, is an inflammatory joint disorder of the axial skeleton [6]. The primary clinical symptom is axial and peripheral enthesitis, an inflammation at the sites of attachment of ligaments, tendons and joint capsules to bone. Ankylosis and joint immobility subsequently develop due to heterotopic cartilage and bone formation. It is still unclear, however, how entheseal inflammation is coupled to ankylosis in AS. Regarding this, it is worth noting that the pathology observed in the axial and peripheral joints of *Dcir*^−/−^ mice closely resembles that of AS in humans. However, the etiopathogenesis seems different, because HLA-B27 and endoplasmic reticulum-stress-induced activation of the IL-23/IL-17 axis are involved in AS but not in the ankylotic changes in *Dcir*^−/−^ mice [6]. Nonetheless, the critical roles of IFN-γ in the development of ankylosis in *Dcir*^−/−^ mice suggest that IFN-γ may also be involved in the development of ankylosis in AS patients.

CLRs are widely recognized as one of pattern recognition receptors that sense pathogen-derived carbohydrate structures and initiate innate and adaptive immune responses against pathogens [1]. Actually, DCIR-Fc fusion protein binds helminthes such as *S. mansoni* and *T. spiralis*. DCIR signaling suppresses TLR8- and TLR9-mediated cytokine productions. Furthermore, HIV-1 binds DCs through DCIR, resulting in the promotion of HIV-1 transmission to CD4^+^ T cells. However, the present report by Maruhashi and Kaifu et al. clearly shows that DCIR is important for the regulation of bone metabolism independently from pathogen infection, suggesting the presence of endogenous ligands [3]. Endogenous ligands are also reported recently for other CLRs such as Mincle, Clec12a, and Clec9a, suggesting that this group of lectins also plays important roles apart from host defense against pathogens [7]. The presence of EPS motif that enables binding to galactose-containing ligands in the carbohydrate recognition domain suggests glycans as the ligands [1]. Identificaiton of DCIR ligans should be important for understanding the molecular mechanisms how DCs differentiation and bone formation are controlled, and may provide us a clue to develop novel therapeutics for bone metabolic disorders.
